# Cocota's Story: Life Lessons in Aging, Resilience, and End‐of‐Life Agency From a Brazilian Matriarch

**DOI:** 10.1111/jgs.19566

**Published:** 2025-06-03

**Authors:** Thiago J. Avelino‐Silva, Niousha Moini

**Affiliations:** ^1^ Division of Geriatrics, School of Medicine University of California San Francisco California USA; ^2^ Laboratorio de Investigacao Medica em Envelhecimento (LIM‐66), Servico de Geriatria, Hospital das Clinicas HCFMUSP, Faculdade de Medicina Universidade de Sao Paulo Sao Paulo Sao Paulo Brazil

**Keywords:** aged, longevity, personal autonomy, resilience

## Abstract

Resilience is increasingly recognized as a central factor in how older adults adapt to life's inevitable changes, yet many clinicians remain unfamiliar with its practical applications in late life. Drawing on the true story of Cocota, a Brazilian matriarch who lived to be 100, this special article illustrates how resilience is neither a static trait nor limited to mere survival. Instead, it emerges over decades, shaped by early adversities and sustained through purposeful roles, strong social ties, and an enduring sense of autonomy. Although psychological, social, and spiritual resources are crucial, physiological resilience also plays an essential role, reflecting adaptive responses at organ, cellular, and molecular levels that help older adults recover from acute health stressors. In Cocota's case, a hip fracture in her 80s did not lead to permanent disability; rather, she reclaimed her daily routines, demonstrating the interplay between physical robustness and unwavering determination. Equally telling was her decision to “stop eating and drinking” near life's end, exemplifying resilience as a final expression of agency. We further explore how her experiences align with deeper forms of well‐being, marked by purpose and prosocial behavior, and practical wisdom, including emotional regulation and sound moral judgment. By examining her life journey, clinicians and community partners can better appreciate how resilience spans physical, cognitive, psychosocial, and spiritual domains, ultimately guiding more integrated strategies to support older adults. The lessons learned have direct relevance for clinical interventions, community programs, and public health initiatives aimed at fostering autonomy and meaningful engagement in later life.

## Graphical abstract

Resilience, cultivated across physical, cognitive, psychosocial, and spiritual domains, shapes how older adults navigate late‐life challenges yet remains unfamiliar to many clinicians. Drawing on the 100‐year journey of Brazilian matriarch Cocota, this article demonstrates how lifelong adaptations, from overcoming early hardships to achieving self‐determination at life's end, provide actionable cues for fostering autonomy and purpose in aging (Adapted from Abadir et al. J Am Geriatr Soc. 2023;71:2381–2392).

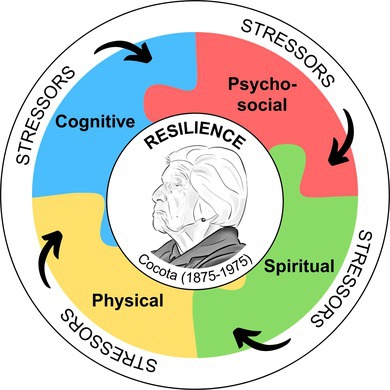

**Summary**

- Key points
  ○ Resilience in late life is built through decades‐long adaptations that integrate physiological and cognitive reserve with psychological, social, and spiritual resources, rather than simply the absence of disease.
  ○ Final acts of agency (including self‐determined end‐of‐life decisions) can embody resilience as the culmination of a lifelong commitment to autonomy, illustrating how older adults integrate bodily limits, personal meaning, and cultural values into their choices.
  ○ Clinicians can nurture resilience by coupling strategies that strengthen physical reserve (e.g., regular exercise, balanced nutrition) with programs that expand social engagement (e.g., intergenerational activities) and emotional coping skills (e.g., reframing, mindfulness), thereby translating resilience theory into geriatric practice.
- Why does this paper matter?
  ○ This case‐based narrative challenges ageist assumptions by showing how multidomain resilience shapes health outcomes and end‐of‐life choices. It offers clinicians, researchers, and care partners a concrete example of how older adults can maintain dignity, independence, and purpose across the lifespan.

## 1 Introduction

Resilience has emerged as a central construct in understanding how older adults adapt to life's inevitable changes [1]. Although the literature spans decades of theoretical models and quantitative measures, many clinicians and practitioners remain less familiar with resilience's practical applications in late life. Indeed, the need to optimize remaining capacities while compensating for age‐related losses has never been greater. By actively cultivating emotional, social, and spiritual supports, and by rebounding from hardships with renewed purpose, older adults can shape their own aging trajectories, maintaining a sense of competence and hope despite adversity [2].

In this special article, we use the real‐life account of a Brazilian matriarch, Cocota, to illustrate how resilience is neither a static trait nor confined to mere survival. Instead, it unfolds as a lifelong process, molded by early adversities (e.g., arranged marriage, childlessness) and culminating in later adaptations that encompass multiple domains: physical recovery from serious injury, long‐lasting cognitive engagement, emotional self‐regulation, intergenerational social bonds, and even spiritual agency at life's end. Our case‐based narrative aligns with contemporary frameworks of aging resilience and advances their understanding by (1) highlighting resilience's integrative nature across the lifespan, (2) showcasing the power of lived experience in countering ageist stereotypes, and (3) reframing resilience to include self‐determined closure as an act of autonomy. Throughout, we hope this antiageist narrative will help inform clinicians and researchers in clinical, psychosocial, and public health aging, thereby strengthening the theoretical and empirical lenses through which resilience in later life is understood.

### 1.1 The Woman Behind the Portrait, as Told by Avelino‐Silva

**FIGURE 1:**
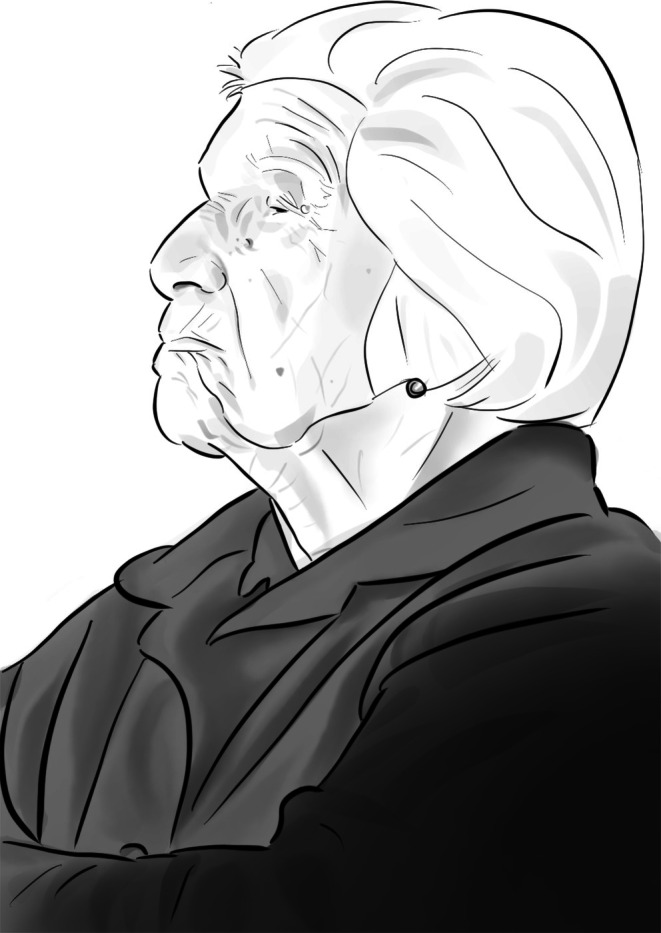
Portrait of Cocota (1875–1975), by Avelino‐Silva. The portrait of Cocota captures her in profile, an elegant simplification of line and shade that conveys both gravitas and softness. The face, etched with the grooves of a long life, carries the weight of countless stories—episodes of strength, loss, humor, and perseverance. The set of her jaw and the curve of her shoulders suggest dignity. The illustration pays tribute to a woman known for stubborn kindness, for outlasting expectations, and for performing quiet acts of courage.

My great‐grandmother Cocota was born in 1875 in Poços de Caldas, a thermal spring town in Minas Gerais, Brazil, during the final years of Emperor Dom Pedro II's reign. The nickname, an affectionate term meaning “graceful little girl,” would soon overshadow her given name and become inseparable from her identity. She lived to be 100, witnessing the twilight of an empire, her country's gradual path to modernity, and the passage of multiple generations within her household. Although she died before I could meet her, her presence always felt both tangible and instructive in our family, as if I had known her personally (Figure 1).

Cocota's early life was shaped by expectations and family decisions beyond her control. Forced into an arranged marriage at 14, she entered adulthood as a child bride, her destiny seemingly circumscribed by tradition. Yet, as years passed, no children arrived. Instead, she carved out a role as a caregiver for ailing relatives and neighbors. Embodying a quiet strength, she could be found at the side of those who were ill or frail, earning a reputation as the family's unwavering anchor. Over the years, she sat vigil at countless sickbeds, tending to people stricken with tuberculosis and later the devastating 1918–1919 influenza pandemic. Remarkably, she remained untouched by either disease. In summary, rather than resigning herself to circumstance, she reshaped it by becoming indispensable to those in need.

Cocota was, in fact, my great‐grandaunt, not my great‐grandmother by blood. But family histories often unfold in unexpected ways, and although she would never bear a child of her own, she did not remain childless. Her sister‐in‐law, Dacica, my actual great‐grandmother, had delivered a fourth child and then fell gravely ill shortly afterward. The attending physician predicted she would not survive. In those critical hours, Dacica summoned Cocota to her bedside. Facing death, she entrusted the newborn to Cocota's care, offering this infant son as her own, binding the two with a covenant born of love and desperation. When the baby's father arrived, he balked at this extraordinary arrangement and demanded the child's return. Cocota firmly refused, declaring, “This one was given to me and is now mine.” Remarkably, Dacica recovered, but the child remained with Cocota, growing up calling two women by maternal titles: “Mãe” (Mother) and “Minha Mãe” (My Mother).

Cocota aged with remarkable resilience, sustained by a strong sense of purpose. After taking on the unexpected role of motherhood, she forged a deep bond with her nephew‐turned‐son. Over time, she became an unfaltering presence in the upbringing of eight grandchildren and later had the joy of sharing her daily life with nearly 20 great‐grandchildren. Even into her 80s, she walked daily to the local grocery. One fateful day, a horse‐drawn cart ran her over, breaking her hip. Taken to São Paulo for surgery, she was met with the blunt assessment of a surgeon who installed plates and screws, then remarked that these would last about a decade—long enough, he implied, since she would likely die before then. She reacted by withdrawing temporarily into herself, remaining bed‐bound with closed eyes, seemingly inert. Family members feared the worst. Days passed. They took her back home, seemingly disabled for life. The moment she set foot on her home's threshold, she opened her eyes, rose from the bed, and resumed daily life as if nothing had happened. Pain and adversity were forces to be acknowledged but not surrendered to. In this manner, she aged with resilience by repeatedly adapting to life's challenges, finding purpose in caregiving despite early adversity, and maintaining both independence and cognitive engagement into her later years. She would ultimately also exercise control over her final moments, embodying the physical, cognitive, psychosocial, and spiritual dimensions of resilience.

Her final decade was one of graceful stubbornness. Mobility became challenging. She struggled to rise from chairs and walk without discomfort. Even so, she refused special treatment or to be fed in her bedroom. She would join everyone at the dinner table, reading the newspaper well into her 90s, her cognition intact, her dignity uncompromised. In her later years, reaching 100 became a milestone to hold onto. When that day finally came, it coincided with my parents' wedding celebration, an event that drew hundreds of friends and relatives, who came as much to congratulate the newlyweds as to honor the century‐old matriarch. The next day, however, she declared, “Agora, chega” (Now, no more). She needed to rest, withdrawing peacefully, refusing meals, moving toward a quiet end. Within 4 months, she passed away at home, leaving a legacy not only of family continuity but of profound human resilience.

### 1.2 Lessons and Reflections on Resilience

Institutionalization, marginalization, and ageist assumptions often render the wisdom and experiences of older adults invisible [3], depriving us of living archives of resilience, adaptability, and nuanced perspectives on the human life course [4]. As global demographics shift, we are increasingly entering what might be termed the “age of longevity.” [5] Still, many modern societies struggle to integrate older adults fully [6], including the oldest‐old who stand at the frontier of these demographic changes. By overlooking their lessons in resilience, we risk perpetuating the very ageist notions that undermine their vital contributions.

**FIGURE 2:**
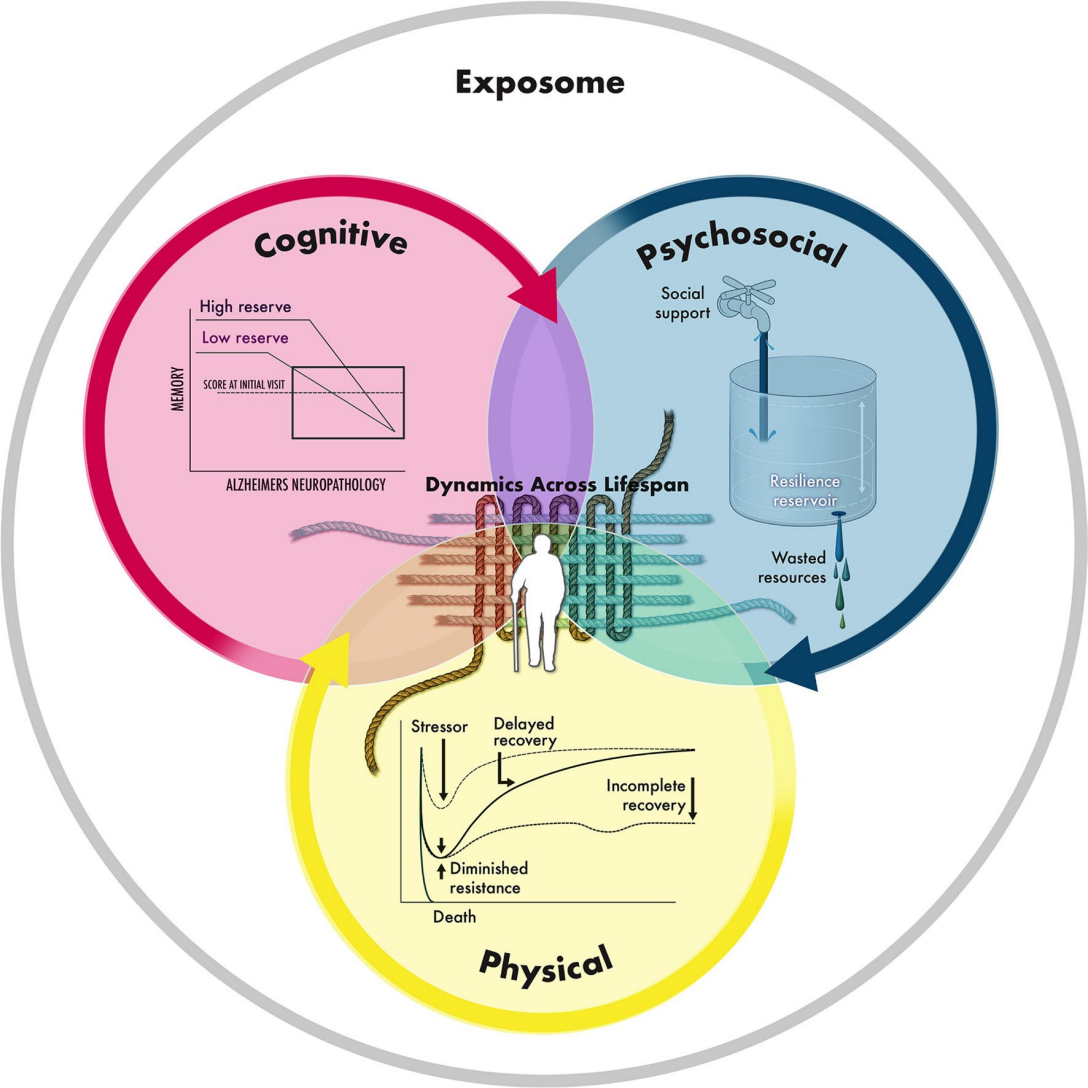
Multidimensional resilience framework across the lifespan. Resilience is portrayed as the woven “warp‐and‐woof” of life, formed by the continuous interaction of three overlapping domains—physical (yellow), cognitive (magenta), and psychosocial (blue)—all nested within the broader exposome (gray ring). The physical inset shows how a stressor can precipitate diminished resistance and delayed or incomplete physiological recovery; the cognitive inset illustrates how higher baseline reserve can buffer memory decline despite Alzheimer neuropathology; and the psychosocial inset depicts a “resilience reservoir” that is replenished by social support or depleted by wasted resources. Together, these strands highlight the cumulative, lifelong interplay of stressors, adaptations, and supports that ultimately shape each individual's resilience trajectory. Reproduced from reference [12].

Resilience in aging involves far more than the mere avoidance of disease or the preservation of physical function [7]. It encompasses cognitive engagement, emotional regulation, social participation, and the capacity to find meaning despite progressive change (Table 1). Contemporary gerontological frameworks conceptualize resilience as multidimensional (Figure 2) [12, 13, 14]: cognitive resilience underpins the capacity to retain or regain mental function despite neurologic or stress‐related insults; psychological resilience supports identity maintenance and well‐being under adversity; social resilience fosters interdependence and mutual support; and spiritual resilience provides a structure for integrating losses and transitions within a coherent life narrative. Notably, spiritual resilience can also be seen as a dimension of wisdom: it elicits deeper insights about human life and nurtures subjective well‐being through compassion, gratitude, and an impulse to enhance the well‐being of others. By framing adversity within a meaningful narrative, older adults are guided not merely toward self‐preservation but toward caring for and improving the lives of others.

**TABLE 1:** Key concepts for resilience in older adults.

Concept	Definition/explanation
Resilience in older people	Set of positive attitudes of older adults, coupled with available resources (e.g., social support, spiritual faith, family ties, personal beliefs, financial means), that enable them to adapt, cope, recover, and even grow when facing adversity (e.g., illness, disability, loss, socioeconomic challenges).
Attributes	Social, emotional, cultural, environmental, or spiritual supports that can be drawn on in times of adversity (e.g., help from family or neighbors, strong faith, economic stability). Adaptive coping strategies, including optimism, sense of purpose, self‐efficacy, perseverance, resolution, fortitude, humor, empathy, and altruism.
Antecedents	Sociodemographic characteristics (e.g., age, education, income). Experiences of adversity (e.g., widowhood, chronic illness, forced migration). Social context (e.g., personal relationships, neighborhood resources). Intrinsic aspects (e.g., spirituality, goals, personal values).
Consequents	Positive outcomes or manifestations that follow resilient responses: Improved mental health and emotional well‐being (fewer depressive symptoms, more effective stress management). Increased quality of life and greater overall life satisfaction. Reduced pain and physical discomfort. Lower incidence of stress‐related illnesses (e.g., cardiovascular disease, immune‐system dysregulation). Strengthened capacities for gratitude and forgiveness. Active aging (retaining independence, engaging in meaningful activity). Optimistic outlook and personal growth. Active aging marked by maintained independence and engagement in meaningful activities. Sustained optimism and ongoing personal growth.
Empirical elements	Methods and instruments used to assess resilience in older adults (e.g., Wagnild and Young Resilience Scale [8]; Connor‐Davidson Resilience Scale [9]; Brief Resilient Coping Scale [10]; Groningen Aging Resilience Inventory [11]; qualitative data exploring how older adults experience and define resilience).
Life‐course perspective	Resilience in later life reflects earlier life experiences, cumulative advantages/disadvantages, and adaptive skills learned over time. Older adults may be “inoculated” by prior adversity or, conversely, experience diminished coping reserves if chronic hardship has persisted.
Implications for practice	Strengthen or tailor resource availability (e.g., enhance social networks, facilitate community engagement, improve financial security). Clinical promotion of resiliency skills (problem‐solving, mindfulness, managing feelings). Foster positive attitudes and coping (e.g., promote counseling for optimism and self‐efficacy). Assess antecedents (e.g., health status, economic conditions) to identify vulnerable older adults early. Use empirical scales and qualitative interviews to evaluate resilience systematically.

Nevertheless, cognitive, psychosocial, and spiritual reserves represent only part of the story. A complementary, though sometimes underemphasized, dimension is physical (or “physiological”) resilience, understood as the ability to withstand or recover from functional decline following acute and/or chronic health stressors [15, 16]. While cognitive and psychological resilience addresses how individuals maintain mental processes and emotional well‐being in adversity [17], physical resilience focuses on the maintenance or regaining of function when faced with clinical challenges such as serious infection, hip fracture, or other acute insults [18]. It presupposes adaptive physiological responses at the organ, cellular, and molecular levels (e.g., musculoskeletal, immunological, neurological) that help older adults preserve homeostasis under demanding conditions [19]. In contrast to frailty, which typically connotes vulnerability and is measured at a single point in time, physical resilience can be evaluated at multiple intervals after a stressor to estimate the trajectory of recovery rather than mere baseline risk [19]. An apt analogy depicts an “old castle under siege,” in which structural integrity, stored resources, and timely repairs collectively determine whether a stressor causes irreparable damage or fosters successful rebound [19, 20]. Older adults show remarkable heterogeneity in their capacity to repair tissue damage, modulate inflammatory pathways, or sustain strength and mobility [21]. Such biologic characteristics interact reciprocally with social, emotional, and behavioral factors (e.g., physical activity, nutrition, community engagement) to shape overall resilience [19, 21].

In other words, resilience in older adulthood is neither an isolated trait nor a fixed state; it emerges from a dynamic, transactional process between individuals and their ever‐changing environments [22]. Resilience depends not only on internal factors like coping skills, self‐efficacy, and a sense of purpose but also on social contexts that shape and sustain it. Viewing resilience solely as a personality trait can inadvertently place blame on individuals who cannot overcome adversity, overlooking the broader socioecological influences that foster or hinder their capacity to adapt. Early adversity, for example, can nurture psychological resources (e.g., coping strategies, emotional regulation, and personal agency) that later serve as anchors when physical function declines. Research indicates that resilience is measurable [8, 9, 10, 11], and older adults with higher resilience tend to experience lower rates of depression and cognitive decline, later onset of disability, better physical rehabilitation outcomes, and reduced healthcare utilization [23]. Furthermore, consistent engagement in meaningful activities reinforces autonomy, cognitive stimulation, and emotional well‐being [24]. By integrating lifelong patterns of challenge, adaptation, and supportive environmental interactions, older adults can navigate late‐life transitions with dignity, agency, and a sustained sense of purpose. In the paragraphs below, we apply these constructs to show how the case of Cocota both aligns with and supports existing resilience frameworks.

Cocota's early experiences navigating arranged marriage, childlessness, and the unexpected responsibility of raising her nephew fostered coping skills, resourcefulness, and emotional fortitude that would serve her well across the life course. Her anecdotal resistance to severe respiratory illnesses foreshadowed the physical resilience that, decades later, allowed her to recover from a hip fracture without embracing a permanently dependent role. Indeed, she returned to most of her usual routines in her 80s, refusing to define herself as “bedridden.” Her steadfast insistence on joining others at the table, reading the newspaper, and engaging in stimulating conversations even in advanced old age demonstrates psychological and cognitive resilience. Meanwhile, her integral role within the extended family network highlights the significance of social connections and a sense of purpose. Such aspects align with well‐established correlates of healthy aging, including sustained engagement in meaningful activities, perceived control, and the maintenance of social ties [25]. Moreover, by adapting to hardship and actively shaping family structures, she ensured that future generations benefited from her purposeful caregiving and intergenerational bonds. In so doing, she modeled each dimension of resilience: physical (defying medical expectations), cognitive (maintaining mental alertness through continuous engagement), psychological (transforming early adversity into meaning), social (forging strong, cross‐generational ties), and spiritual (her final act of self‐determination). These interconnected forms of resilience not only fortified her own identity and agency but also wove a lasting legacy of resourcefulness and solidarity, illustrating how “profound human resilience” can extend beyond one individual to uplift an entire social network.

Cocota's trajectory also illustrates how resilience intersects with broader constructs of well‐being and wisdom, particularly in older adulthood. By devoting herself to caregiving and maintaining a sense of meaning and connection, she exemplifies a form of eudaimonic well‐being centered on purpose, autonomy, and personal growth [26]. Her ability to navigate complex moral and social dilemmas, from adopting her nephew as a son to engaging in self‐determined end‐of‐life decisions, reflects core elements of practical wisdom, including emotional regulation, sound judgment, and prosocial behavior [4]. Rather than simply enduring adversity, she integrated it into a narrative of self‐transcendence, thereby demonstrating how resilience, well‐being, and wisdom can converge to enrich both individual and family trajectories across the lifespan.

There are also practical implications to strengthening resilience (Table 1). Clinicians and community partners can bolster resilience in older adults by jointly targeting physical, social, and psychological factors [27]. For instance, regular physical activity and preventive home visits both enhance physical capacities and reduce frailty [27]. Equally important is social engagement through community‐based activities, volunteering, and leadership initiatives that foster shared decision‐making and collective agency [27, 28]. On a psychological level, group interventions (e.g., savoring, gratitude, and meaning‐based tasks) and positive mental health practices (e.g., reframing, belonging, purpose) have been shown to reduce perceived stress and improve mental well‐being [28]. Integrated approaches that combine these strategies, addressing physical, social, and psychological domains in unison, can achieve a synergistic effect on resilience, especially when caregiver support is also accounted for [25].

To complete this continuum of care, teams can embed brief, strengths‐oriented counseling that cultivates optimism, gratitude, and self‐efficacy, psychological assets associated with slower functional decline and better survival [29]. Additionally, “resilience checklists” can flag red‐flag antecedents such as multimorbidity, loneliness, or food insecurity and trigger tiered referrals to social work, nutrition, or home‐support services [27]. Finally, routine tracking with validated tools and periodic life‐story interviews allows clinicians to individualize goals and adjust supports in real time [12]. This closed‐loop approach—cultivate mind‐set, safeguard resources, monitor risk, and measure response—can operationalize resilience strengthening at the bedside and in the community, enabling older adults to navigate uncertainty, pain, and loss with greater autonomy and meaning.

However, resilience in late life does not imply infinite perseverance. Near the end, when Cocota decided she had “had enough,” she engaged in a behavior that can be understood through the lens of autonomy, end‐of‐life decision‐making, and what literature sometimes terms “voluntarily stopping eating and drinking.” [30] While on the surface this may seem like resignation, from the gerontological and bioethical perspectives, it can be viewed as a deliberate, agency‐driven act. As individuals approach the limits of functional capacity and personal meaning, choosing the timing and conditions of one's own death can reflect control and self‐determination. Such a decision, while appearing as withdrawal, may be recognized as the culmination of resilience—a final assertion of personal dignity and coherence in the face of mortality. In sum, resilience in aging transcends mere survival. It resides in the capacity to adapt, remain engaged, preserve dignity, and ultimately navigate the end of life on one's own terms.

Cocota's story grounds key findings from resilience research in a concrete, lived example. First, it illustrates how comprehensive frameworks of aging resilience can be observed in practice, spanning early‐life adversity, mid‐life role adaptation, and self‐determined end‐of‐life decisions. Second, the narrative reveals how physical, cognitive, psychosocial, and spiritual dimensions of resilience manifest as an integrative lifelong process rather than isolated traits. Third, it translates abstract constructs into concrete behaviors that clinicians can identify, assess, and strengthen. Recognizing the value of older adults, listening to their stories, and respecting their final decisions broadens our understanding of what it means to age well. Cocota's century‐long life and the quiet strength she embodied—captured gracefully in a portrait—remind us that resilience is an ongoing negotiation with the forces of change, a set of skills and dispositions honed across a lifetime, and, at its conclusion, an opportunity to claim authorship over one's final narrative. By sharing a glimpse of her life, we hope to inspire clinicians, researchers, and care partners to help cultivate and sustain resilience across the lifespan.

## Author Contributions

All authors contributed to the manuscript concept, drafting, and revision. Thiago J. Avelino‐Silva created the portrait figure. All authors approved the final version of the manuscript for submission.

## Disclosure

Sponsors had no role in the conception, writing, or decision to submit this manuscript for publication.

## Conflicts of Interest

The authors declare no conflicts of interest.
